# Supplementary material for: Nse5/6 is a negative regulator of the ATPase activity of the Smc5/6 complex
Source: Nucleic Acids Res. 2021 Apr 13;49(8):4534–49. doi: 10.1093/nar/gkab234 (PMC8096239; doi:10.1093/nar/gkab234)
Supplement: gkab234_Supplemental_File [file gkab234_supplemental_file.pdf]

SUPPLEMENTARY FIGURE 1

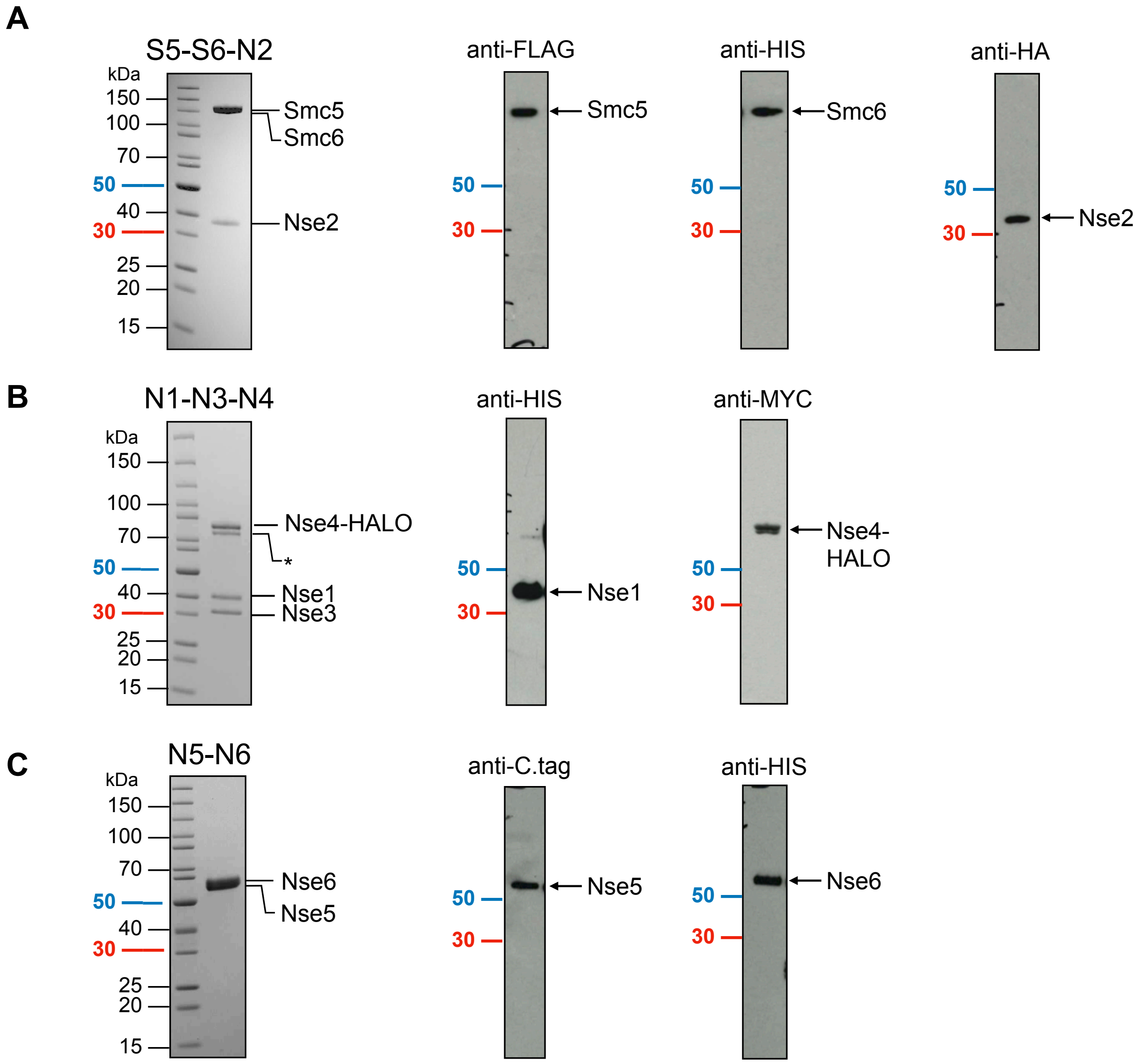

Figure S1. Confirmation of protein identity and migration position on SDS/PAGE gels by western blot

Representative colloidal-blue stained SDS-PAGE gel (left) and associated western blots (right) for each of the purified sub-complexes, expressed by the indicated recombinant baculovirus. (**A**) S5-S6-N2. (**B**) N1-N3-N4. (**C**) N5-N6. The epitope recognised by the primary antibody in each western blot is indicated. To aid comparison, the migration position of the 50 (coloured blue) and 30 kDa (red) molecular mass markers is also highlighted.

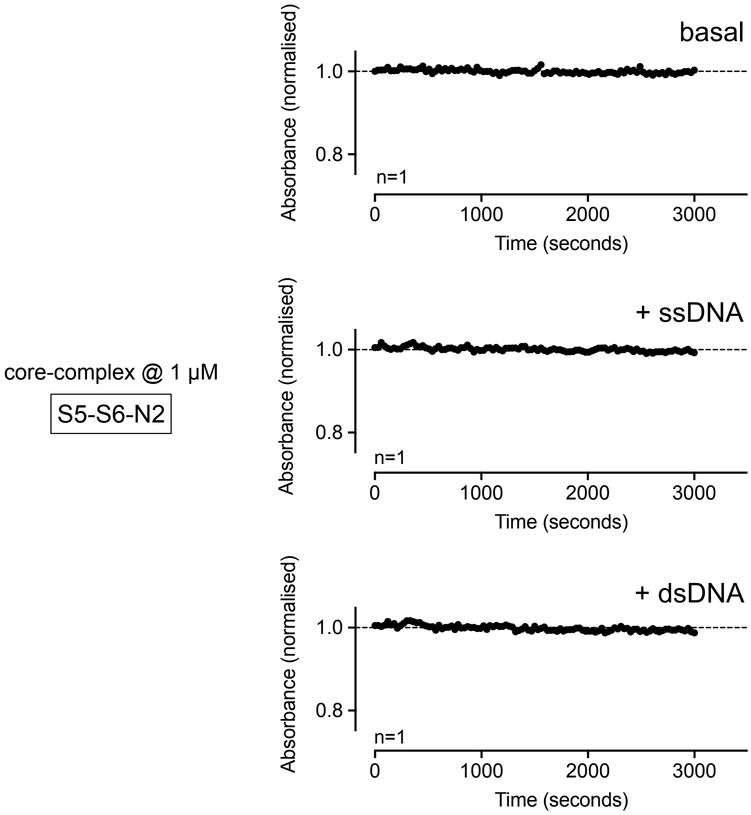
core-complex @ 1 μM
S5-S6-N2
basal
Absorbance (normalised)
1.0
0.8
n=1
0
1000
2000
3000
Time (seconds)
+ ssDNA
Absorbance (normalised)
1.0
0.8
n=1
0
1000
2000
3000
Time (seconds)
+ dsDNA
Absorbance (normalised)
1.0
0.8
n=1
0
1000
2000
3000
Time (seconds)

Figure S2. The Smc5/6 'core-complex' does not turnover ATP

Purified Smc5/6 'core' complex, comprising Smc5, Smc6 and Nse2, does not turn over ATP in a NADH-coupled regenerative ATPase assay, even when tested at a final concentration of 1 μM (7-fold higher than the data presented in Figure 3B). Addition of either ssDNA or dsDNA still has no stimulatory effect.

Nse5.C-tag / Nse6

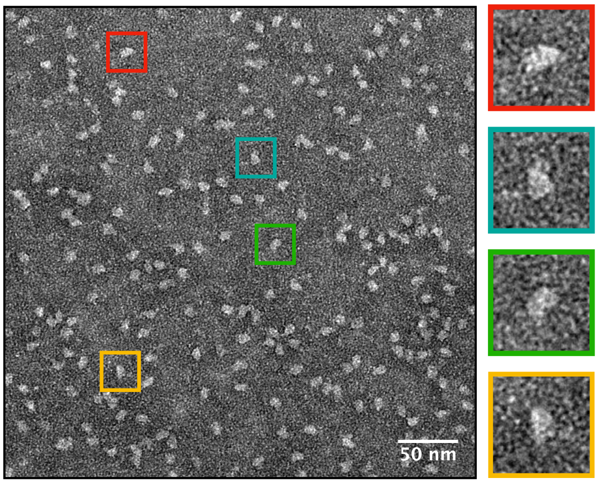

Figure S3. Visualisation of the Nse5/6 heterodimer

Representative micrograph showing particles of purified Nse5/6 heterodimer, negatively stained by uranyl acetate. Selected particles (as indicated by boxes with different coloured borders) are shown at increased magnification on the right-hand side.

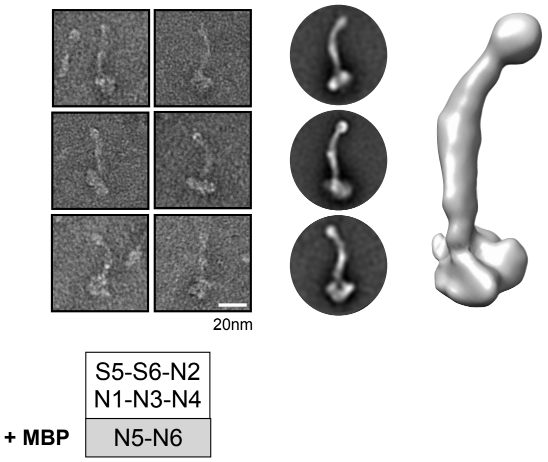
20nm
S5-S6-N2
N1-N3-N4
+ MBP
N5-N6

Figure S4. 2D class averages and 3D model of the Smc5/6 ‘super-complex’ containing Nse5 fused to MBP

(Left) Representative images of individual particles. (Middle) 2D class averages (Right) Initial 3D reconstruction.
